# Supplementary figures and images for: Profiling of terminating ribosomes reveals translational control at stop codons
Source: eLife. 2026 Jul 7;14:RP109257. doi: 10.7554/eLife.109257 (PMC13341110; doi:10.7554/eLife.109257)

Figure 2-Figure Supplement 1

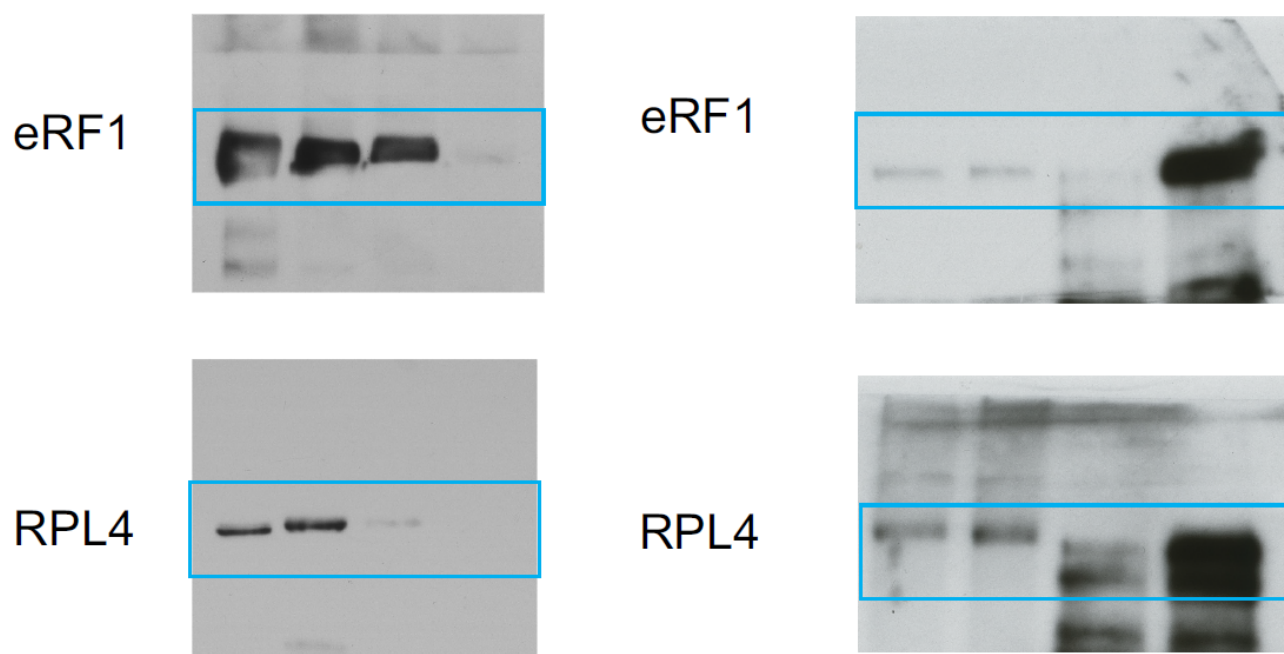

Supplement: Figure 2—figure supplement 1—source data 1. [file elife-109257-fig2-figsupp1-data1.zip › Figure 2-Figure Supplement 1-Source Data 1.pdf]

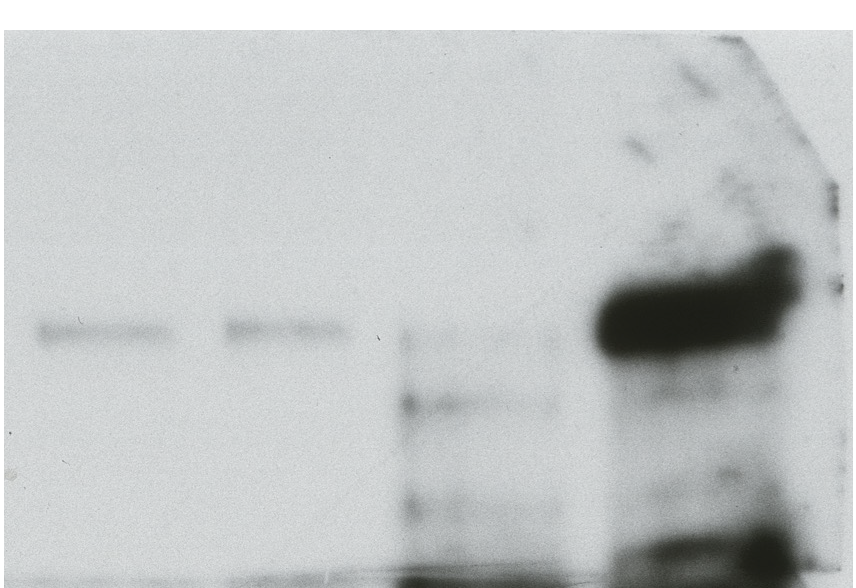

Supplement: Figure 2—figure supplement 1—source data 2. [file elife-109257-fig2-figsupp1-data2.zip › Fig 2 Supplement 1/Fig. 2 Supplement 1 E eRF1 right.tiff]

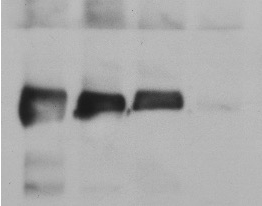

Supplement: Figure 2—figure supplement 1—source data 2. [file elife-109257-fig2-figsupp1-data2.zip › Fig 2 Supplement 1/Fig. 2 Supplement 1 eRF1 left.tiff]

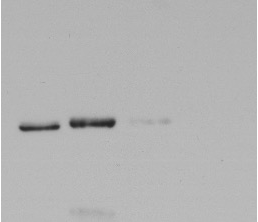

Supplement: Figure 2—figure supplement 1—source data 2. [file elife-109257-fig2-figsupp1-data2.zip › Fig 2 Supplement 1/Fig. 2 Supplement 1 RPL4 left.tiff]

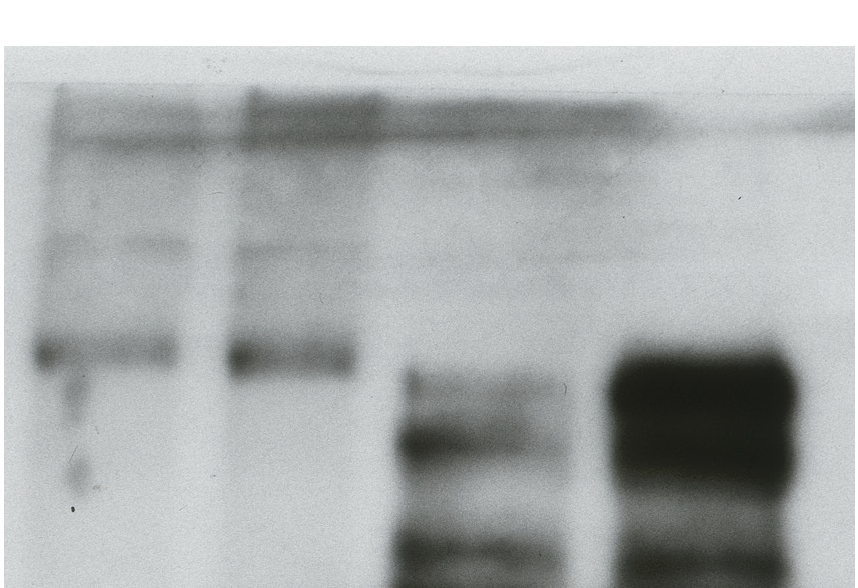

Supplement: Figure 2—figure supplement 1—source data 2. [file elife-109257-fig2-figsupp1-data2.zip › Fig 2 Supplement 1/Fig. 2 Supplement 1 RPL4 right.tiff]

Fig. 4E

HiBiT

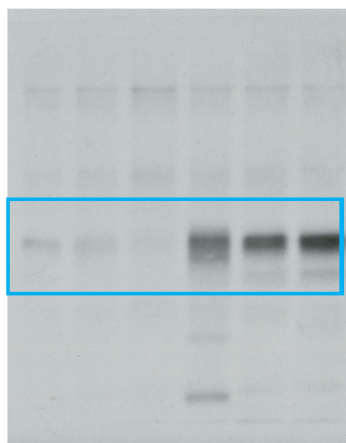

$\beta$ -actin

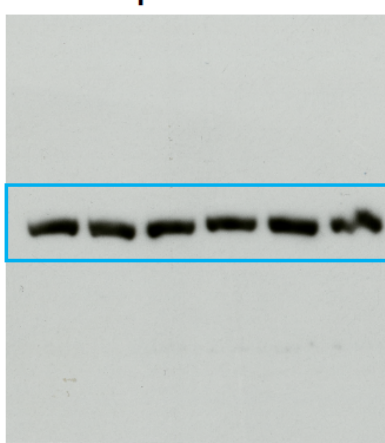

GFP

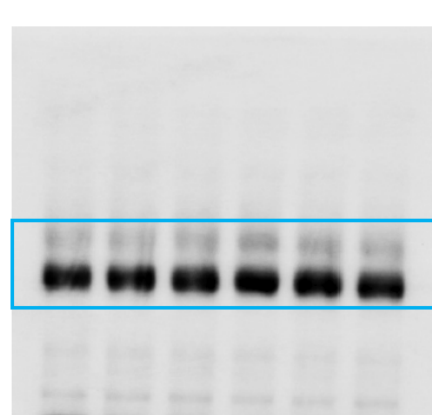

Supplement: Figure 4—source data 1. [file elife-109257-fig4-data1.zip › Figure 4-Source Data 1.pdf]

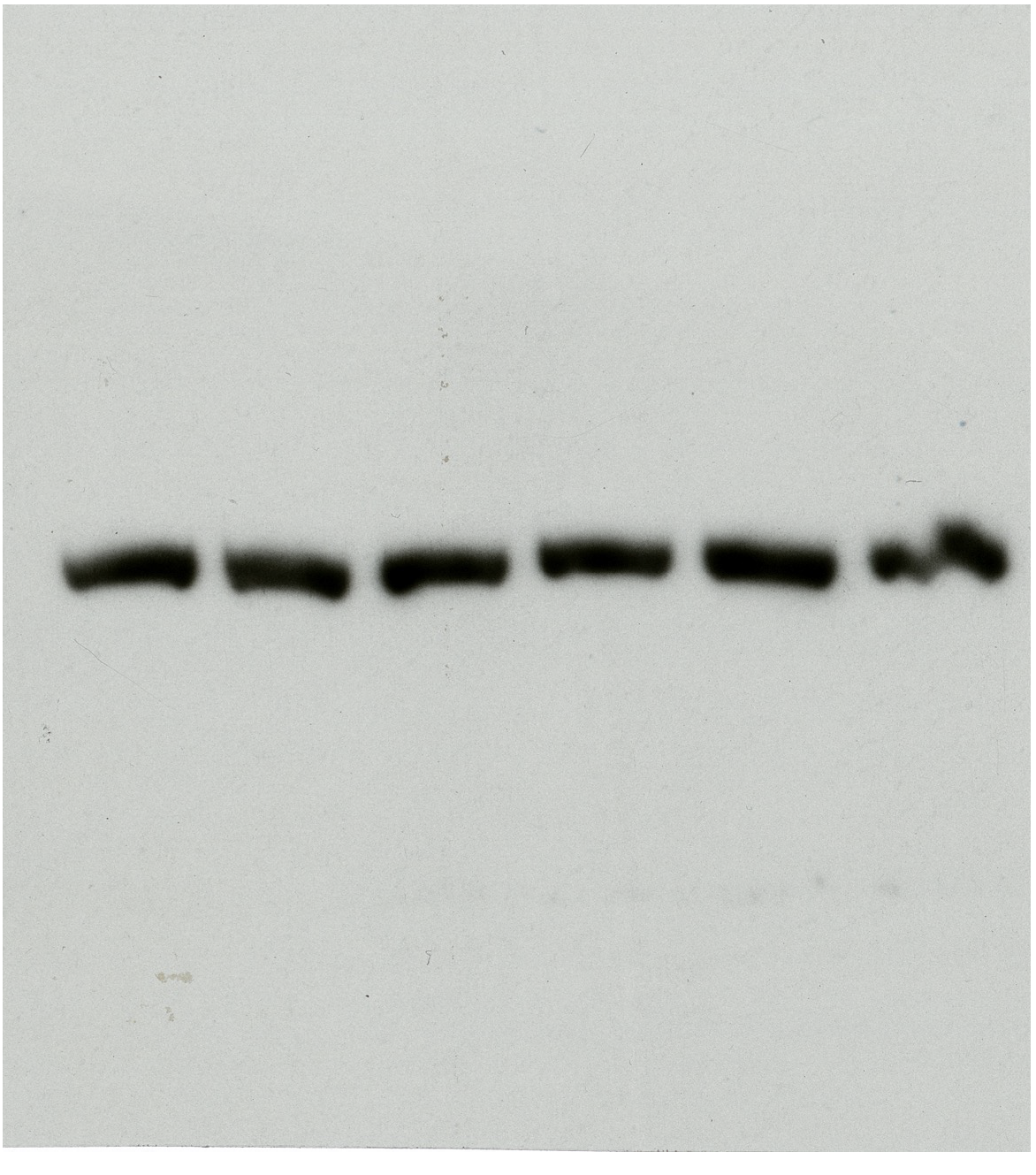

Supplement: Figure 4—source data 2. [file elife-109257-fig4-data2.zip › Fig 4/Fig. 4E beta-actin.tiff]

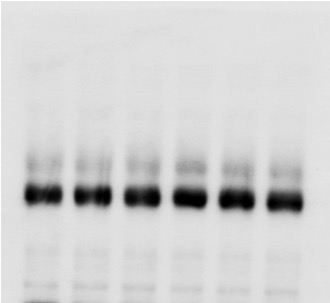

Supplement: Figure 4—source data 2. [file elife-109257-fig4-data2.zip › Fig 4/Fig. 4E GFP.tiff]

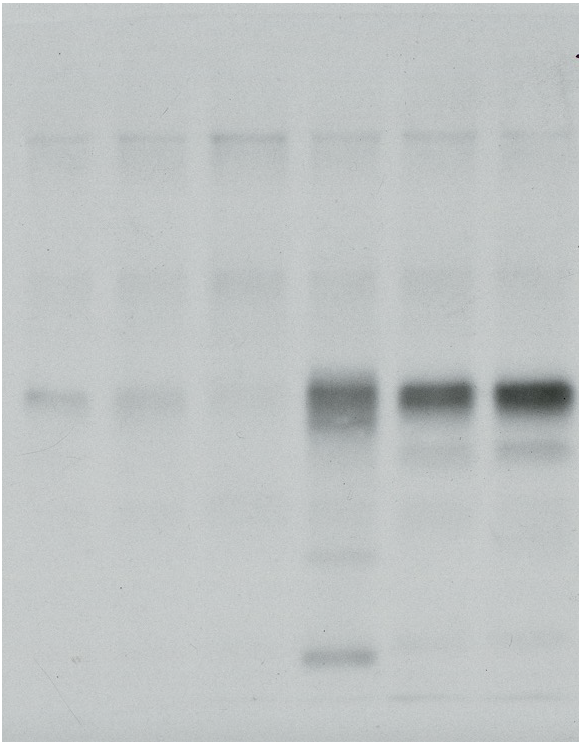

Supplement: Figure 4—source data 2. [file elife-109257-fig4-data2.zip › Fig 4/Fig. 4E HiBiT.tiff]

Figure 4-Figure Supplement 3

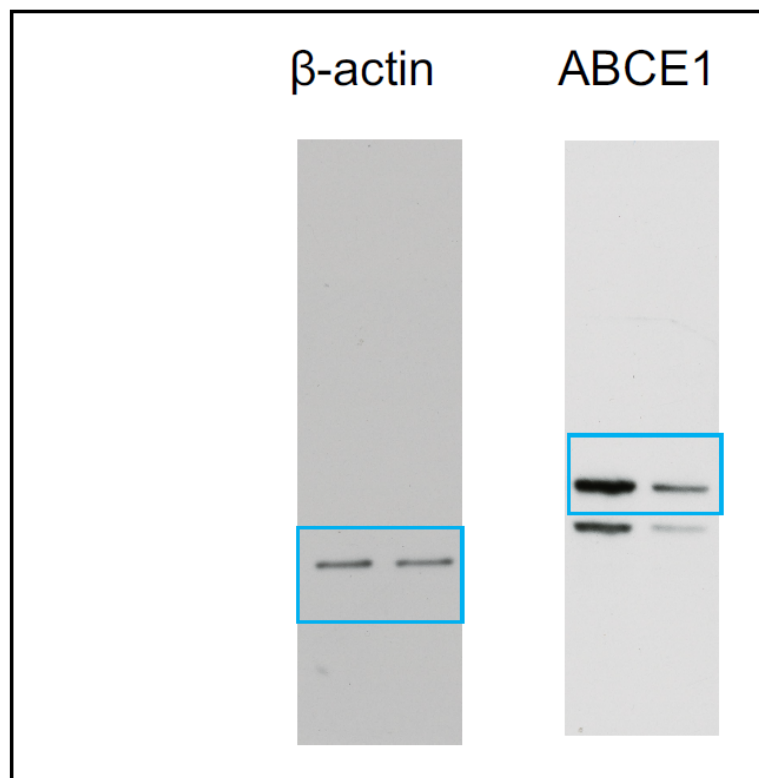

Supplement: Figure 4—figure supplement 3—source data 1. [file elife-109257-fig4-figsupp3-data1.zip › Figure 4-Figure Supplement 3-Source Data 1.pdf]

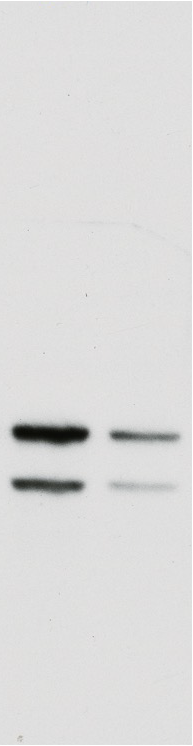

Supplement: Figure 4—figure supplement 3—source data 2. [file elife-109257-fig4-figsupp3-data2.zip › Fig 4 Supplement 3/Fig. 4 Supplement 3 ABCE1.tiff]

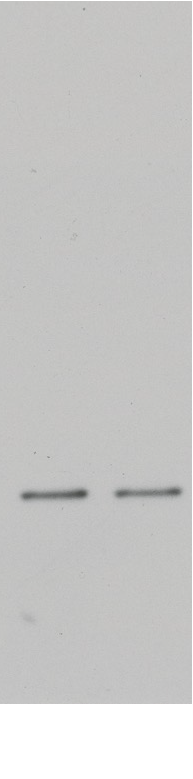

Supplement: Figure 4—figure supplement 3—source data 2. [file elife-109257-fig4-figsupp3-data2.zip › Fig 4 Supplement 3/Fig. 4 Supplement 3 beta-actin.tiff]

Fig. 6B

eRF1

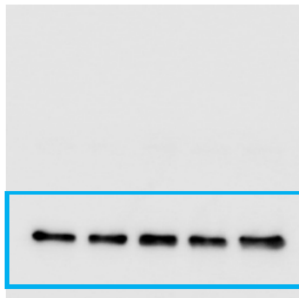

Rps26

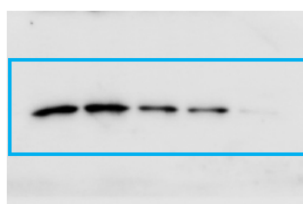

Rpl4

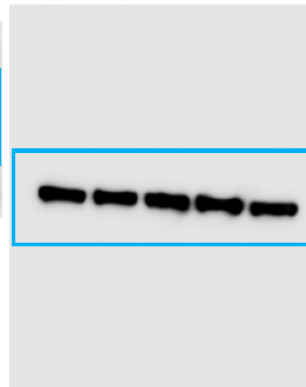

$\beta$ -actin

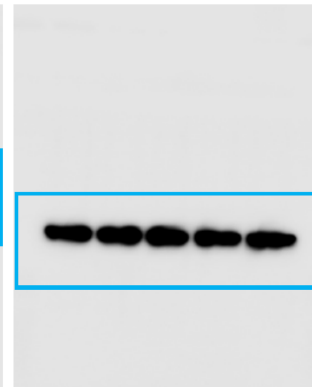

Supplement: Figure 6—source data 1. [file elife-109257-fig6-data1.zip › Figure 6 Source Data/Figure 6-Source Data 1.pdf]

## Liver

eRF3

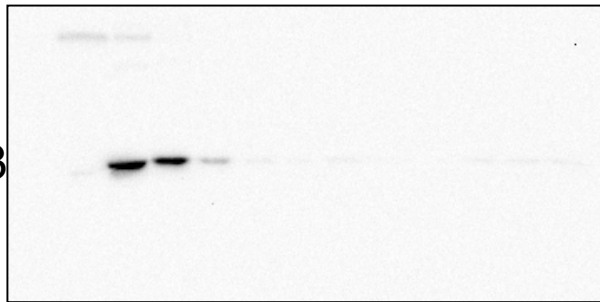

eRF1

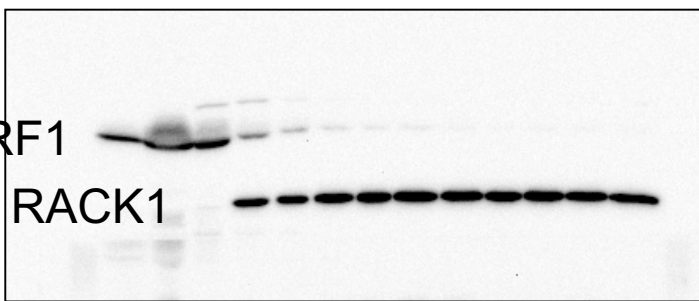

RACK1

Rps26

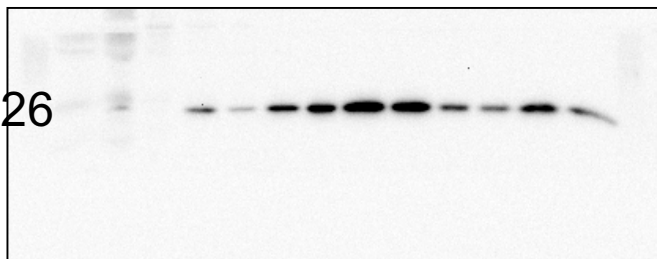

## Testis

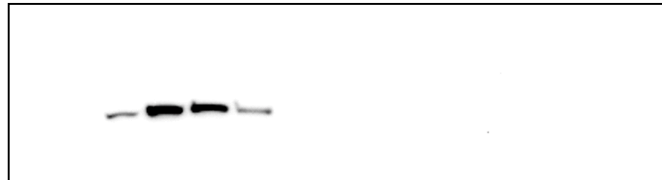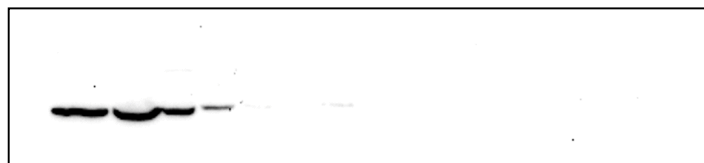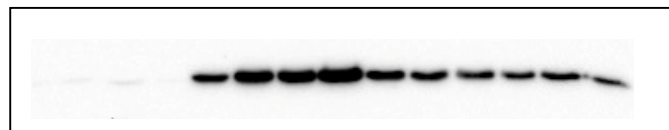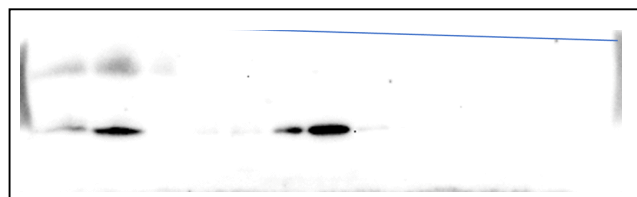

Supplement: Figure 6—source data 1. [file elife-109257-fig6-data1.zip › Figure 6 Source Data/Figure 6-Source Data 2.pdf]

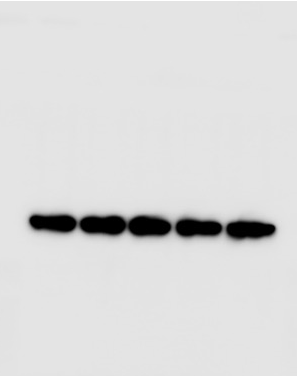

Supplement: Figure 6—source data 2. [file elife-109257-fig6-data2.zip › Fig 6/Fig. 6B beta-actin.tiff]

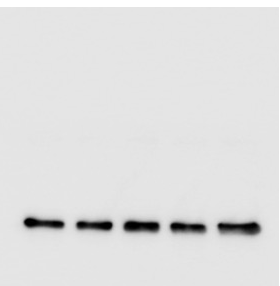

Supplement: Figure 6—source data 2. [file elife-109257-fig6-data2.zip › Fig 6/Fig. 6B eRF1.tiff]

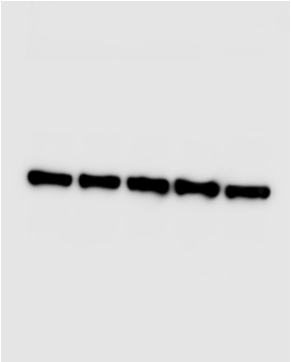

Supplement: Figure 6—source data 2. [file elife-109257-fig6-data2.zip › Fig 6/Fig. 6B Rpl4.tiff]

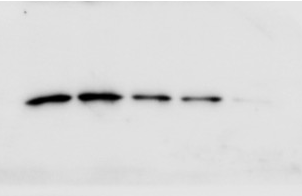

Supplement: Figure 6—source data 2. [file elife-109257-fig6-data2.zip › Fig 6/Fig. 6B Rps26.tiff]

Figure 7-Figure Supplement 1

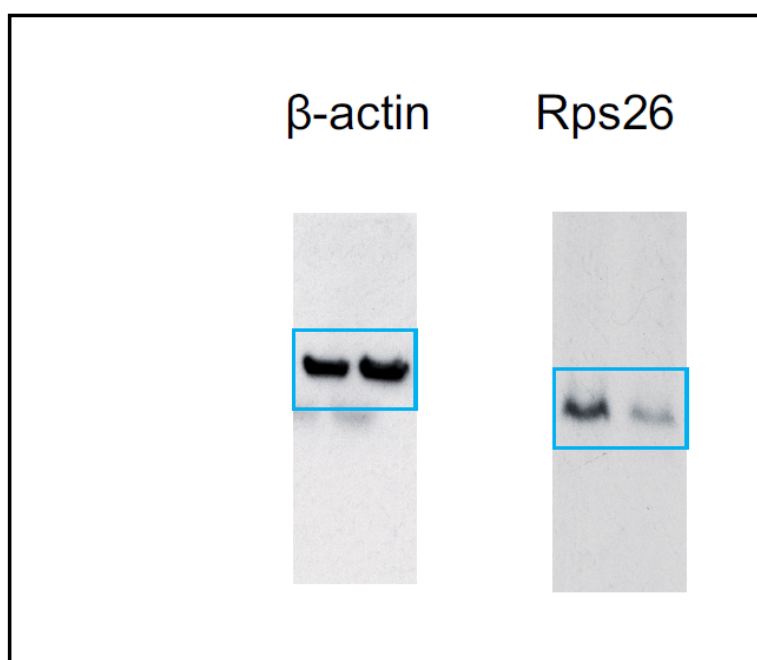

Supplement: Figure 7—figure supplement 1—source data 1. [file elife-109257-fig7-figsupp1-data1.zip › Figure 7-Figure Supplement 1-Source Data 1.pdf]

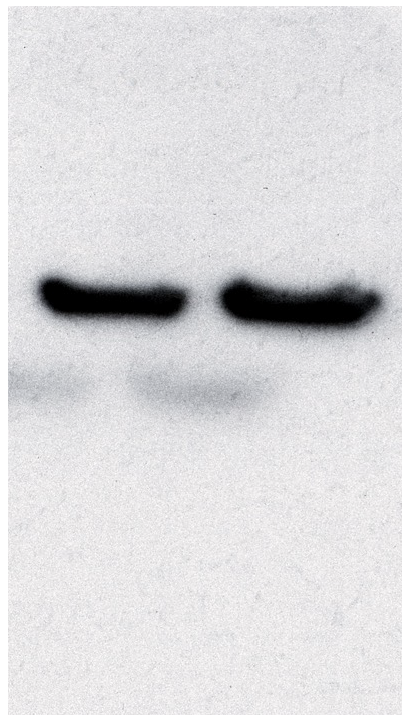

Supplement: Figure 7—figure supplement 1—source data 2. [file elife-109257-fig7-figsupp1-data2.zip › Fig 7 Supplement 1/Fig. 7 Supplement 1 beta-actin.tiff]

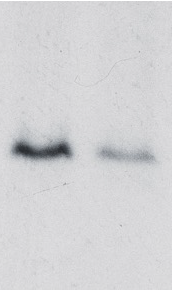

Supplement: Figure 7—figure supplement 1—source data 2. [file elife-109257-fig7-figsupp1-data2.zip › Fig 7 Supplement 1/Fig. 7 Supplement 1 Rps26.tiff]

Figure 7-Figure Supplement 2

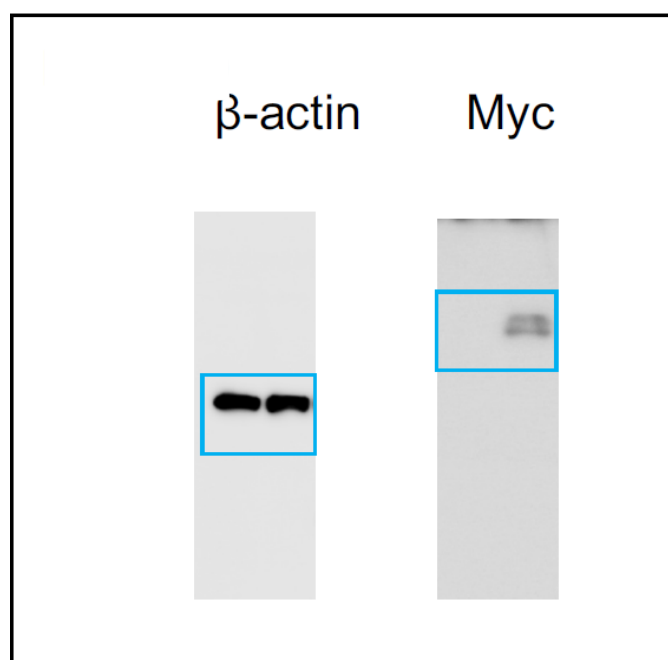

Supplement: Figure 7—figure supplement 1—source data 3. [file elife-109257-fig7-figsupp1-data3.zip › Figure 7-Figure Supplement 2-Source Data 1.pdf]

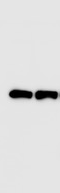

Supplement: Figure 7—figure supplement 1—source data 4. [file elife-109257-fig7-figsupp1-data4.zip › Fig 7 Supplement 2/Fig. 7 Supplement 2 beta-actin.tiff]

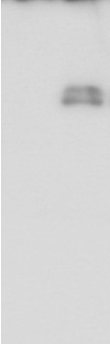

Supplement: Figure 7—figure supplement 1—source data 4. [file elife-109257-fig7-figsupp1-data4.zip › Fig 7 Supplement 2/Fig. 7 Supplement 2 Myc.tiff]
