# Supplementary material for: Profiling of terminating ribosomes reveals translational control at stop codons
Source: eLife. 2026 Jul 7;14:RP109257. doi: 10.7554/eLife.109257 (PMC13341110; doi:10.7554/eLife.109257)
Supplement: Peer review history

# Editor report: eLife Assessment

**DOI:** 10.7554/eLife.109257.3.sa0

**Authors:** Daniel Arango

**Affiliations:** Northwestern University United States

**Keywords:** Compelling, Useful

This manuscript reports on the application of ribosome profiling (EZRA-seq and eRF1-seq) and massively parallel reporter assays (MPRA) to identify and characterize sequence elements that regulate translation termination. The authors conclude that a GA-rich element upstream of stop codons is associated with ribosome pausing during translation termination; in contrast, C-rich sequences upstream of stop codons abolish termination pausing. While the overall findings of this study are **useful** and the identification of GA-rich elements upstream of stop codons is **compelling**, support for several other claims remains incomplete. Specifically, the evidence that the MPRA results mirror the ribosome profiling, that a C-rich sequence preceding the stop codon promotes termination slippage in cellular mRNAs, and that Rps26 interferes with mRNA interactions to regulate translation termination would benefit from further support.

# Referee report: Reviewer #2 (Public review)

**DOI:** 10.7554/eLife.109257.3.sa1

Summary:

This paper presents results interpreted to indicate that sequences upstream of stop codons capable of base-pairing with the 3' end of 18S rRNA prolong the dwell time of 80S ribosomes at stop codons in a manner impeded by Rps26 in the 40S subunit exit channel, which leads to the proper completion of termination and ribosome recycling and prevents spurious translation of 3'UTR sequences by one or more unconventional mechanisms.

Strengths:

The standard 80S and selective eRF1 80S ribosome profiling data obtained using EZRA-Seq are of high quality, allowing the authors to detect an enrichment for purine-rich sequences upstream of stop codons at sites where termination is relatively slow and ribosomal complexes are paused with eRF1 still engaged in the A site.

Weaknesses:

There are many weaknesses in the experimental design and interpretation of results that undermine several of the final conclusions of the study described in the abstract, as described in detail below.

(1) It's not indicated how far upstream of the stop codon the sequences were searched to find the enriched motifs in Figs. 1C and 2D. If it's further upstream of -15 then the sequence would generally not be found in the exit channel of a terminating ribosome positioned with the stop codon in the A site in the manner expected from their final model of mRNA:18S rRNA pairing. (This would be analogous to the occurrence of the Shine-Dalgarno within 15 nt of the initiation codon for most mRNAs in *E. coli*.) They could have depicted nucleotide percentages at each nucleotide from -1 to -15 for the high and low pause stop codons to better facilitate consideration of their proposed mechanism of termination pausing involving the 3' end of 18S rRNA.

(2) lines 234-242: Their reporter data in Fig. 4B suggest that only the presence of GGG triplets at any location in the 9 nt substantially prevents downstream translation. If their interpretation about these G-rich sequences promoting termination by forming G-quadruplexes is correct, then this would have little to do with the purine-rich motifs identified by the profiling experiments (and their proposed function in base-pairing with rRNA), as the purine-rich motifs do not feature GG bases (as shown in Fig. 2D in particular). The authors point out that the MPRA can sample sequence space not represented in living cells. While true, this doesn't change the fact that it failed identify sequences conforming to the purine rich motifs found by the profiling experiments and identified instead sequences capable of forming G-quadruplexes that may well function by a different mechanism than that employed in cells. The authors cannot persist in claiming that the MPRA results confirm the findings of the profiling experiments regarding the purine-rich motif. Also, the claim of enrichment for C-rich sequences in the MPRA results is not compelling as only 3 of the 11 triplets showing the smallest M/P ratios contain more than 1 C and three of them contain no Cs. Also, there was no evidence for depletion of C's upstream of the stop codons with low pause scores from the ribosome profiling data in Fig. 1, so it's inaccurate to claim "mirroring" of results from the ribosome profiling and MPRA data on this point as well.

(3) lines 256-260: I still contend that the different results shown in Fig. 4E for the C-rich and GA-rich sequences are not compelling as results for only a single sequence of each type are shown, which might not be typical of the entire class. In fact, the GA-rich sequence has two GG's and could form a G-quadruplex, whereas the GA-rich motifs identified by ribosome profiling and eRF1-seq do not exhibit consecutive GGs, such that the single G-rich sequence chosen for analysis might function by G-quadruplex mediated stalling rather than base-pairing with the 3' end of 18S rRNA, as they actually suggested in their rebuttal. Even the second GA-rich sequence analyzed in Fig. S3G has two GGs. Thus, while the results in Fig. 4 provide support for the notion that C-rich sequences preceding the stop codon promote stop codon read-through, it's important to note that no evidence was obtained by ribosome-profiling in Fig. 1 that the increased 3'UTR translation seen for low-pause stop codons is associated with C-rich sequences. It's unclear why they would be unable to observe this in the manner they document for the eRF1-Seq data in Fig. 2D for the three C-rich triplets enriched at stop codons lacking eRF1 peaks.

- lines 278-282: These differences are quite small and could arise from the different sequences of the GFP-HiBit fusion proteins, as observed in Fig. 4C (top two control constructs), precluding mechanistic interpretations.

(4) Notwithstanding their claim in the rebuttal, I still find no definition of the GA-rich and C-rich mRNAs described in Fig. 5C in the Methods or legends, nor whether the compilation is restricted to -15 from the stop codons. In addition, if expression of the mutant 18S rRNA is sufficient to alter the height of the termination peaks as shown in Fig. 5C and to alter reporter expression in Fig. 5D, I see no reason why they cannot carry out the pause score/motif enrichment of Fig. 1C to determine if they see the expected diminished enrichment for the GA-motif shown there on expressing the mutant 18S vs. the WT 18S control strain. If not, this would undermine their interpretation of the results in Figs. 5C-D as favoring base-pairing between the 3' end of 18S rRNA and sequences upstream of the stop codon.

(5) I still find a significant shortcoming in their failure to analyze the 18S rRNA 3' end biochemically to show that the expected ~15% with the mutant sequence. Stating simply that they followed a previous protocol is not sufficient to document their success in this notoriously challenging experimental approach.

(6) lines 382-384: The level of the control protein RACK1 is diminished in testis polysomes, and it's unclear that the ratio of Rps26:RACK1 is actually lower in testis polysomes in the manner claimed.

(7) lines 414-427: I still contend that the authors should have quantified the ratio of the stop codon peak to the adjacent coding sequences in Figures 7E to establish that Rps26 OE decreased the stop codon peaks selectively on the GA-rich cohort of mRNAs. In addition, they still have not explained why the C-rich reporter behaves like the GA-rich reporter in Fig. 7F in showing reduced HiBiT expression on Rps26 OE when it should be unaffected. As such, the reporter data do not support the conclusion reached from the data in Fig. 7E.

(8) Notwithstanding their rebuttal I still contend that the failure to measure Rps26 association with 80S ribsoomes or polysomes and show that it is depleted by the shRNA knockdown and increased by Rps26 OE is a significant shortcoming, especially since their interpretation of the OE data depends on the occurrence of 40S subunits lacking Rps26 in unstressed WT cells, which seems improbable based on the prior work on yeast.

(9) Overall, examining the claims in the revised Abstract, I feel that I am in agreement with the claim "We identify a sequence motif upstream of the stop codon that promotes termination pausing,.." but disagree that the function of this motif was "validated by massively paralleled reporter assays", for the reasons stated above in point 2. Regarding the statement "Unexpectedly, reduced termination pausing increases the likelihood of stop codon slippage, giving rise to proteins with heterogenous C-terminal extensions." , I believe it would be more cautious to say that "reduced pausing is associated with stop codon read-through accompanied by frameshifting" since the MRPA did not provide compelling evidence for causality for the reasons described in point 3 above. Regarding the statement "Mechanistically, we show that sequence-dependent termination pausing arises from post-decoding mRNA scanning by the 3' end of 18S rRNA", I find this statement too strong in view of the shortcomings described above in points 4-5 and think it would be more correct to say that their findings are consistent with (rather than showing) this point, and also think they should add qualifying statements to the manuscript acknowledging the limitations of these experiments. I further contend that there are shortcomings in the experiments leading to the conclusion that the stoichiometry of Rps26... modulates mRNA:rRNA interactions, described above in points 6-9. Finally, in the last sentence, the claims that termination pausing is shaped by ribosome heterogeneity, and cell type-specific translational control is too strong.

# Referee report: Reviewer #3 (Public review)

**DOI:** 10.7554/eLife.109257.3.sa2

Summary:

This study from Jia et al carried out a variety of analyses of terminating ribosomes, including the development of eRF1-seq to map termination sites, identification of a GA-rich motif that promotes ribosome pausing, characterization of tissue-specific termination dynamics, and elucidation of the regulatory roles of 18S rRNA and RPS26. Overall, the study is thoughtfully designed, and its biological conclusions are well supported by complementary experiments. The tools and datasets generated provide valuable resources for researchers investigating the mechanisms of RNA translation.

Strengths:

(1) The study introduces eRF1-seq, a novel approach for mapping translation termination sites, providing a methodological advance for studying ribosome termination.

(2) Through integrative bioinformatic analyses and complementary MPRA experiments, the authors demonstrate that GA-rich motifs promote ribosome pausing at termination sites and reveal possible regulatory roles of 18S rRNA in this process.

(3) The study characterizes tissue-specific ribosome termination dynamics, showing that the testis exhibits stronger ribosome pausing at stop codons compared to other tissues. Follow-up experiments suggest that RPS26 may contribute to this tissue specificity.

Weaknesses:

The biological significance of ribosome pausing regulation at translation termination sites or of translational readthrough, for example across different tissue types, remains unclear. Nevertheless, this question lies beyond the primary scope of the current study.

Comments on the latest version:

The authors addressed my comments by revising the claims in the manuscript.

# Author comment: Author response

**DOI:** 10.7554/eLife.109257.3.sa3

**Authors:** Longfei Jia, Yuanhui Mao, Saori Uematsu, Xinyi Ashley Liu, Leiming Dong, Leonardo Henrique França de Lima, Shu-Bing Qian

**Affiliations:** Cornell University Ithaca United States; Cornell University Ithaca United States; Cornell University Ithaca United States; Cornell University Ithaca United States; Cornell University Ithaca United States; Universidade Federal de São João Del Rei Sete Lagoas Brazil; Cornell University Ithaca United States

The following is the authors’ response to the original reviews.

We thank the Editor and Reviewers for their careful evaluation of our manuscript and for the constructive feedback. We agree with eLife’s overall assessment that, while profiling terminating ribosomes provides important insights into termination dynamics, additional clarification of the underlying mechanisms was needed. In response, we have focused our revision on three major conceptual points:

(1) We have moderated our interpretation regarding the contribution of putative mRNA:rRNA interactions to sequence-specific termination pausing and clarified the limitations of the current evidence.

(2) We have refined and clarified our model for the role of Rps26 in regulating translation termination.

(3) We have expanded and strengthened the discussion of tissue-specific termination pausing, including its potential implications and current uncertainties.

**Public Reviews:**

**Reviewer #1 (Public review):**

Summary:

The authors use high-resolution ribosome profiling (Ezra-seq) and eRF1 pulldown-based ribosome profiling (eRF1-seq) developed in their lab to identify a GA rich sequence motif located upstream of the stop codon responsible for translation termination pausing. They then perform a massively parallel assay with randomly generated sequences to further characterize this motif. Using mouse tissues, they show that termination pausing signatures can be tissue-specific. They use a series of published ribosome structures and 18S rRNA mutants, and eS26 knockdown experiments to propose that the GA rich sequence interacts with the 3′-end of the 18S rRNA.

Strengths:

(1) Robust ribosome profiling data and clear analyses clarify the subtle behavior of terminating ribosomes near the stop codon.

(2) Novel termination or "false termination" sites revealed by eRF1-seq in the 5′-UTR, 3′-UTR, and CDS highlight a previously underappreciated facet of translation dynamics.

Weakness:

(1) Modest effects seen in ABCE1 knockdown do not seem to add up to the level of regulation. The authors state "ABCE1 regulates terminating ribosomes independent of the sequence context" on pg 9, and "ABCE1 modulates termination pausing independent of the mRNA sequence context" in the figure caption for Figure S4. Given the modest effect of the knockdown, such phrasing is most likely not supported. Further clarification of "ABCE1 plays a generic role in translation termination" is necessary.

We acknowledge that the modest effects observed for ABCE1 are likely influenced by incomplete knockdown in HEK293 cells. Importantly, the increased ribosome density occurred at all stop codons rather than in a sequence-dependent manner, supporting the conclusion that ABCE1 functions broadly in termination rather than acting in a sequence-specific context. We have revised the manuscript to clarify this point and to temper our interpretation accordingly.

(2) The authors propose that the GA rich sequence element upstream of the stop codon on the mRNA could potentially base pair with the 3′-end of the 18S rRNA. In the PDBs the authors reference in their paper and also in 3JAG, 3JAH, 3JAI (structures of terminating ribosomes with the stop codon in the A-site and eRF1), the mRNA exiting the ribosome and the 3′-end of the 18S rRNA are about 25-30 A apart. In addition, a segment of eS26 is wedged in between these two RNA segments. This reviewer noted this arrangement in a random sampling of 5 other PDBs of mammalian and human ribosome 80S structures. How do the authors anticipate the base pairing they have proposed to occur in light of these steric hindrances? RpsS26 is known to be released by Tsr2 in yeast during very specific stresses. Is it their expectation that termination pausing in human/mammalian cells happens during stressful conditions only?

We agree that structural rearrangements in the absence of Rps26 remain speculative. In the revised manuscript, we have removed overly definitive language and clarified that, while Rps26 dissociation has been reported under stress conditions, its stoichiometry is unlikely to be exclusively stress-dependent. We now present this aspect as a working model supported by indirect evidence rather than a demonstrated structural mechanism.

(3) The authors say, "It is thus likely that mRNA undergoes post-decoding scanning by 18S rRNA." (pg. 10). It is unclear what the authors mean by "scanning." Do they mean that the mRNA gets scanned in a manner similar to scanning during initiation? There is no evidence presented to support that particular conclusion.

We appreciate the comment regarding the term “18S rRNA scanning.” We recognize that this wording may have been misleading and have revised the relevant text to more accurately describe post-decoding mRNA–rRNA interactions without implying an active scanning mechanism.

(4) Role of termination pausing in the testis is highly speculative. The authors state: "It is thus conceivable that the wide range of ribosome density at stop codons in testis facilitates functional division of ribosome occupancy beyond the coding region." It is unclear what type of functional division they are referring to.

We agree that the functional significance of testis-specific termination dynamics remains unclear. As multiple reviewers raised this concern, we have substantially expanded the discussion of tissue-specific termination pausing, explicitly outlining current limitations and framing this as an important direction for future investigation.

**Reviewer #2 (Public review):**

Summary:

This paper presents results interpreted to indicate that sequences upstream of stop codons capable of base-pairing with the 3' end of 18S rRNA prolong the dwell time of 80S ribosomes at stop codons in a manner impeded by Rps26 in the 40S subunit exit channel, which leads to the proper completion of termination and ribosome recycling and prevents spurious translation of 3'UTR sequences by one or more unconventional mechanisms.

Strengths:

The standard 80S and selective eRF1 80S ribosome profiling data obtained using EZRA-Seq are of high quality, allowing the authors to detect an enrichment for purine-rich sequences upstream of stop codons at sites where termination is relatively slow and ribosomal complexes are paused with eRF1 still engaged in the A site.

Weaknesses:

There are many weaknesses in the experimental design, interpretation of results, and description of assay design and assumptions, the data obtained, and the interpretation of results, all of which detract from the scientific quality and significance of this work. In fact, a large proportion of paragraphs in the text and figure panels present some difficulty either in understanding how the experiment or data analysis was conducted or what the authors wish to conclude from the results, or that stem from an overinterpretation of findings or failure to consider other equally likely explanations.

We appreciate the reviewer’s thoughtful evaluation and constructive suggestions. We recognize that our original description of the MPRA and reporter assay results may have lacked sufficient clarity, particularly regarding the sequence motifs associated with termination pausing. In the revised manuscript, we have carefully rewritten these sections to clarify the experimental design, data interpretation, and relationship between sequence context and termination dynamics. We believe these revisions address the reviewer’s concerns and improve the overall clarity of the manuscript.

**Reviewer #3 (Public review):**

Summary:

This study from Jia et al carried out a variety of analyses of terminating ribosomes, including the development of eRF1-seq to map termination sites, identification of a GA-rich motif that promotes ribosome pausing, characterization of tissue-specific termination dynamics, and elucidation of the regulatory roles of 18S rRNA and RPS26. Overall, the study is thoughtfully designed, and its biological conclusions are well supported by complementary experiments. The tools and datasets generated provide valuable resources for researchers investigating the mechanisms of RNA translation.

Strengths:

(1) The study introduces eRF1-seq, a novel approach for mapping translation termination sites, providing a methodological advance for studying ribosome termination.

(2) Through integrative bioinformatic analyses and complementary MPRA experiments, the authors demonstrate that GA-rich motifs promote ribosome pausing at termination sites and reveal possible regulatory roles of 18S rRNA in this process.

(3) The study characterizes tissue-specific ribosome termination dynamics, showing that the testis exhibits stronger ribosome pausing at stop codons compared to other tissues. Follow-up experiments suggest that RPS26 may contribute to this tissue specificity.

Weaknesses:

The biological significance of ribosome pausing regulation at translation termination sites or of translational readthrough, for example, across different tissue types, remains unclear. Nevertheless, this question lies beyond the primary scope of the current study.

We thank the reviewer for the positive assessment of our work. We agree that tissue-specific differences in termination pausing were insufficiently described in the original submission. In response, and in light of similar concerns from other reviewers, we have expanded the relevant sections in the main text and Discussion. We now more clearly articulate both the biological context and the current limitations, identifying tissue-specific regulation of termination as an open question and future research direction.

**Reviewer #4 (Public review):**

Summary:

This manuscript by Qian and colleagues utilizes ribosome profiling, and reporter assays to dissect translation termination. Unfortunately, the data do not support the conclusions of the paper, controls are missing and several assays are not well validated and do not reproduce previous findings from others.

Specific comments:

Translation termination has been studied in several organisms including mammalian cells and yeast. In those cases what is analyzed is not the peak height at the stop codon, but rather the difference in the ribosome density before and after the stop. Thus, analyzing peak height is not validated. I understand that this is relevant only for the ribosome profiling experiments (and Ezra-seq) not the RF1 profiling. But much of the data was acquired that way.

Moreover, the data do not reproduce previous findings and no effort is made to connect them to previous data. Previous data has shown that stop codon efficacy varies. This is not reproduced (S1C). Similarly, an effect from the +1 residue is not reproduced. The data isn't even stratified by different stop codons as previous work has shown that different surrounding residues have different effects in the context of different stop codons. Thus, none of the sequencing data is validated or trusted and does not reproduce previous findings.

The GA-rich sequence identified by Ezra-Seq and RF1 seq is not the same and it differs from previous sequences (Wangen &Green).

The authors claim that the majority of Rf1 peaks is at stop codons, but that is not true. It is only about 30% of the peaks. Also, not all mRNAs have peaks at the stop codons. That is at best problematic. Finally, there are mRNAs that are known to "suffer" from NMD, what do these look like in the Ezra-Seq and RF1-Seq? How about mRNAs that have programmed frameshifts? This raises questions on the validity of the eRF1 data.

Figure 4: First, instead of M/P ratio, one should analyze M/M+P, to normalize out differences in the loading and effects from collisions, which are guaranteed to occur here, but not considered or analyzed. Second, the data are analyzed as if what matters are codons in the P and E site (and beyond, where there are definitely NOT recognized codons). While there is evidence for some interactions, one would think that an additional analysis based on sequence would be helpful. Also, the supplemental data indicates that very rarely are there reciprocal changes (as should be the case), and as seen for stop codons.

Regarding the HiBit reporter assay: The two sequecnes clearly have effects on translation without considering stop codon context (Figure 4C), which need to be taken into account. Also, the effect from the sequences varies in the context of the assay in 4C and 4D (2-fold vs .5 fold), further questioning the assay. Moreover, the authors claim that re-initiation cannot account for Hibit levels, but that is clearly incorrect. The western in Figure 4E does not reproduce the data in 4D. While Hibit goes up as in 4D, the putative GFP-fusion goes down. Finally, while the second reading frame should be more efficient is not explained and further argues for an artifact. Previous work (and work herein) suggests that read-through occurs equally in each reading frame. No controls for these assays are presented: e.g. stimulation by antibiotics, ABCE1 depletion, etc.

Figure 5 has similar problems. I don't understand how the Figure in 5A is made, but when you overlay the cited structures on Rps26, the molecules are identical. I guess the authors used some fantasy to build non-existing sequences differently into the structure. There is no basis for that. In panel C and the same in Figure 7, the number of analyzed mRNAs varies. This could influence the outcome and the EXACT same set of mRNAs should be analyzed. But the main problem here is that the authors need to analyze readthrough and not peak height as detailed above. Essential controls are missing that show what fraction of the 18S rRNA is mutated. Previous work has shown that 2 nt truncated 18S rRNA is actively degraded. It is hard to believe how 15% of altered ribosomes can abolish 100% of the effect from the C-rich sequences. Important validation is missing: the authors should analyze rRNA sequences in their ribo-seq dataset to demonstrate that they have the mutated rRNAs, and that these enrich and de-enrich as predicted.

In Figure 5-7 the authors develop a model that the sequence selectivity arises from base pairing between 18S rRNA and the mRNA. If so, then they should really stratify the data by number of WC pairs that can be formed. And only WC pairs, as GU pairs have a totally different geometry that will likely be discriminated against in this context. Also, the mutation is in a part of the helix that has no effect (Figure S3G). Thus, the data within the manuscript are inconsistent.

Figure 6 does not agree with published data (Li et al., Nature 2022). Previous work did not show testis-depletion of Rps26 in purified ribosomes. This is the critical difference as the authors here did not purify ribosomes. Also, another Rps is an essential control, even if purified ribosomes are used. The validity of this dataset is thus questionable . Depletion from polysomes is hard to believe, as overall there is less signal in the polysomes.

Figure 7 has similar problems as figure 5. Different pools of mRNAs are analyzed; peak height is not validated. Overexpression of Rps26 is not shown, as only Myc is shown, not Rps26. Beyond that, increased occupancy in ribosomes needs to be shown for the effect to come from ribosomes. Given how sick the cells are it is most likely that all effects are secondary and arise from whatever else is going on in the overexpression or depletion of Rps26. No controls are presented to show specific effects from Rps26.

The authors need to check Rli1/ABCE levels in their cells. Their data have features that are indicative of low ABCE1 levels. These include a very small effect from ABCE1 depletion. These could be responsible for some of the effects they observe.

We appreciate the reviewer’s engagement with our study and the opportunity to clarify several points.

With respect to perceived inconsistencies with prior literature, we emphasize that our findings do not contradict established principles of translation termination. Rather, enabled by the development of eRF1-seq, we provide higher-resolution insight into termination dynamics that extends existing models. We have revised the manuscript to better contextualize our findings within prior studies and to avoid overstating novelty where continuity exists.

Regarding the analysis of ribosome profiling data, we note that peak height and read density are widely used metrics for inferring ribosome dwell time and pausing. Nevertheless, we recognize that our original presentation may not have sufficiently explained this analytical framework. In the revised manuscript, we have clarified the rationale and interpretation of peak-based analyses, particularly in Figures 5 and 7 involving 18S rRNA mutants and Rps26 perturbation.

Finally, we appreciate the reviewer’s comments concerning base pairing. We have carefully revised both the Results and Discussion sections to present mRNA–rRNA interactions as a supported but not definitively proven mechanistic model, clearly distinguishing experimental evidence from inference.

We are grateful for the reviewers’ thoughtful feedback. We believe the revisions have strengthened the manuscript by clarifying interpretations, moderating mechanistic claims, and expanding discussion of tissue-specific regulation, while preserving the central contributions of the study.

**Recommendations for the authors:**

**Reviewer #1 (Recommendations for the authors):**

(1) Some minor typos are present in the main text and methods section.

We thank the Reviewer’s attention to detail in reviewing our manuscript. We have now thoroughly revised the main text and methods section.

(2) S1I is missing or unlabelled.

We are glad to have this opportunity to fix this mistake. Both S1I and S5D have now been added to the revised figures.

(3) Could the authors clarify in the main text whether crosslinking was a step in the eRF1-seq protocol? Pg 5: "Without crosslinking, ribosomal proteins were minimally pulled down by the eRF1 antibody, confirming the transient nature of eRF1 binding."

Yes, crosslinking is needed for eRF1-seq. We tried no-crosslinking but very little was pulled down, as stated in the sentence in Page 5.

(4) Are termination events in the 5′-UTR or the CDS, as seen in the eRF1-seq data, also influenced by the GA-rich sequence? If the data is disaggregated into those two buckets, can you still pull out the motif?

Yes, stop codons in 5’UTR and CDS share the same feature. However, the number of 5’UTR stop codons captured by eRF1-seq are too few to generate reliable sequence motif analysis.

(5) Could the authors please clarify what peaks/fractions they are using as the monosome in Figure 4A? From the manner in which the red boxes are drawn on the sucrose gradient profile traces, it seems that the 40S, 60S, 80S monosome and half of the disome peak are included in the monosome fraction.

The red box shown in Figure 4A is a bit misleading. For the massive paralleled reporter assay, we selected ribosome fractions based on the sucrose gradient tracing corresponding to monosome and polysomes, respectively. However, the fraction accuracy is not absolute as the fraction tube corresponding to monosome could contain traces of subunits as well as disomes. In practice, 40S and 60S are less concerned than disome, but the primary component is 80S ribosome.

(6) On page 13, please cite references for Normal mode analysis.

Normal Mode Analysis (NMA) using the Anisotropic Network Model (ANM) is a computationally efficient method for predicting large-scale, functional, and directional protein motions near equilibrium. We have followed the Reviewer’s suggestion by citing a review paper in the field of structural biology (Bahar, I. et al. 2005).

**Reviewer #2 (Recommendations for the authors):**

(1) The authors interpret the height of RPF peaks at stop codons in their Ribo-Seq data as an indication of pausing by ribosomes during termination, resulting from slow or inefficient decoding of the stop codon and peptide release; although it could equally result from slow recycling of the 60S subunit by ABCE1 following peptide release. Arguing against the latter possibility, they show later in the study that shRNA knockdown of ABCE1 has little effect on the stop codon RPF peaks; however, because the ABCE1 depletion does not elicit collisions near the stop codon in the manner observed in other studies, it appears that the ABCE1 depletion was insufficient to impair recycling substantially. The authors also don't attempt to support their interpretation by showing that depletion of eRF1 increases the stop codon peaks and produces collisions just upstream of the stop codon. They never specify with any precision whether it is stop codon recognition by eRF1, peptide hydrolysis, or recycling of the 60S subunit from the post-termination complex that is delayed, which is very unsatisfying.

We agree with the Reviewer that the RPF density at stop codons only reflects the dwell time of terminating ribosomes. In fact, it is not possible to dissect molecular details from Ribo-seq data sets, same as interpreting other pausing events. Regarding ABCE1, we did observe the increased termination peak in cells with ABCE1 knockdown (Figure S4C). The lack of collisions is perhaps due to incomplete depletion of ABCE1. Notably, ABCE1 depletion selectively increased ribosome density at the –15 nt position, whereas the forward-shifted –12 nt peak was largely unaffected (Figure S4D). These results suggest that ABCE1 primarily facilitates late-stage termination or ribosome splitting, and its absence delays pre-termination progression. Nevertheless, the main focus of the study is to decipher the sequence context of termination pausing, which seems to be irrelevant to ABCE1. We thank the Reviewer for understanding.

(2) They found enrichment for a GA-rich motif in the mRNAs with the largest stop codon peaks, which they attribute to its effect in slowing down some aspect of termination or ribosome recycling to increase the dwell time of the terminating ribosomes. They found no motif, however, in mRNAs containing the smallest RPF peaks at stop codon peaks, which presumably terminate more rapidly; even though they conclude later in the study from their massively parallel reporter assays (MPRA) that "C-richness" in the 9 nt 5' of stop codons enables rapid termination. The mRNAs with high pause scores at the stop codon that are enriched for the GA motif also show lower RPFs in 3'UTRs compared to the low pause score mRNAs, which they interpret to mean that long-lived termination complexes produce more efficient peptide termination and ribosome recycling, while short-lived complexes fail to be recycled and continue translation into the 3'UTR. However, because the 3'UTR reads are in all three frames, this could not occur simply by stop codon readthrough but would also require a frameshift upstream or at the stop codon itself to prevent termination and continued translation into the 3'UTR; and it could also arise from unconventional reinitiation by unrecycled post-termination complexes, which has been seen by others on inhibition of 60S recycling. The authors' interpretation is too simplistic.

We thank the Reviewer’s summary about the sequence features controlling ribosome dwell time at stop codons uncovered by eRF1-seq. We are fully aware of the complex scenarios about 3’UTR translation, however, unconventional reinitiation cannot explain the results of the reporter assay shown in Figure 4D. Unlike frameshifting that generates prolonged products with mixed C-termini, reinitiation is associated with a new start. In Figure 4D, we observed products with C-terminal fused HiBiT, which cannot be explained by reinitiation. We thank the Reviewer for understanding.

(3) They obtain support for the role of a GA-motif in pausing at stop codons from their selective ribosome profiling of eRF1-bound 80S ribosomes present at stop codons, finding a related GA-motif enriched at stop codons with high occupancies of eRF1-bound RPFs. However, once again, there is no C-rich motif enriched upstream of stop codons with low eRF1-bound RPF occupancies, at odds with later claims for such a motif. They ultimately propose that the GA motif pauses terminating ribosomes by base-pairing with the 3' end of 18S rRNA in the ribosome mRNA exit channel, principally utilizing two UU residues at the penultimate bases in the 18S rRNA that presumably base-pair with either A or G residues in the GA motif.

The Reviewer might be confused by the results from Ribo-seq and massively paralleled reporter assay (MPRA). Ribo-seq data sets are limited to endogenous sequences that were shaped during evolution. In contrast, MPRA uses completely randomized sequences that offer unbiased analysis of sequence elements. The lack of C-rich motif in eRF1-seq data sets is due to the under-representation of such sequence elements in human genome. Perhaps this sequence bias is beneficial for termination fidelity by minimizing 3’UTR translation. We have further clarified this point in the revised manuscript.

(4) They claim to have obtained independent confirmation of this last idea from their massively parallel reporter analysis (MPRA), in which sequences upstream of the stop codon of a uORF were randomized to determine those that appear to prevent translation downstream of the uORF and thereby place the mRNA in the monosome fraction versus those that allow downstream translation by any mechanism including leaky scanning of the uORF start codon, stop codon readthrough, or reinitiation (the assay doesn't distinguish between these mechanisms) and place the mRNA in the polysome fraction. In actuality, their results showed that the presence of only GGG triplets at any location in the 9 nt substantially prevents downstream translation, whereas only CCG and CCC proline codons enable downstream translation by one or more mechanisms. In view of their final model, it's very difficult to understand why GGG at any position would be able to base-pair with the U-U residues in the 18S rRNA when the stop codon is in the A site, and also why the many other triplets with two G's, two A's or an A and G base-all consistent with the GA-rich motif identified earlier-would not act similarly. Similarly, it's also puzzling that CCG and CCC can exert their effects at multiple positions upstream of the stop codon, and why the 7 other codons with two C's do not act similarly. Thus, it's unconvincing that a specific C-rich motif (which they refer to repeatedly but never identify) or even C-richness upstream of the stop codon confers elevated downstream translation. It's also important to note that the MPRA does not report on pausing at stop codons explicitly, only on whether ribosomes can be found downstream of the uORF stop codon, and assigning this outcome to the presence or absence of pausing during termination requires an ad hoc assumption that the authors have not identified as such.

The Reviewer brought up excellent points in this comment regarding the MPRA result. Indeed, MPRA does not report ribosome pausing events as pointed out by the Reviewer. Additionally, MPRA is not designed to distinguish mechanisms underlying translational readthrough. As we mentioned above, both MPRA and Ribo-seq bear different experimental features that partly explain the similar, but not identical sequence motif uncovered by two assays. The prominent GGG motif identified by MPRA is intriguing, reminiscent of our prior study focusing on translation initiation (Jia et al. NSMB 2020). We propose that G-rich sequences upstream of stop codons form G-quadruplexes that block ribosome movement, resulting in monosome enrichment. Supporting this notion, the GGG motif was not identified by eRF1-seq, echoing the importance of using complementary experimental procedures in drawing conclusions.

(5) They claim to confirm their conclusions from the profiling and MPRA data by measuring translation of the HiBiT sequence inserted downstream of the stop codon of the uORF in two reporters in which the upstream 9 nt contain either a single C-rich sequence or a single G-rich sequence. It's unclear how or why these two particular sequences were chosen. The G-rich sequence does not conform closely to either of the GA-motifs captured in the sequence LOGOs of Figures 1-2, and as noted above, there was no C-rich motif ever identified in these analyses. Thus, it's unclear whether the different effects of these two sequences are representative of sequences that pause or do not pause terminating ribosomes that they identified by the genome-wide analyses. In addition, given that the exact position of the GG or CC sequences relative to the stop codon doesn't seem to matter based on the MPRA data, it is actually possible to find the same number of base pairs with the 3' end of 18S rRNA for both of the two GA-rich and C-rich sequences analyzed in these reporter assays by sampling different registers of pairing between the mRNA and 18S rRNA. What is needed instead is be a systematic analysis using both the polysome:monosome assay, and the HiBiT translation assay of sequences that can pair perfectly with the 18S rRNA or contain increasing numbers of mismatches predicted to destabilize the putative helix that would be formed, and to determine whether the stability of the helices thus formed is highly correlated with the presence of the reporter mRNA in monosomes and with low HiBiT translation.

We appreciate the Reviewer’s effort to improve our manuscript. The sequences inserted into the reporters were chosen based on several considerations. First, we chose the GA-motif rather than the G-rich sequences because the former represents physiological sequence element uncovered by eRF1-seq. As mentioned above, the G-rich sequences could form G-quadruplex artifacts. Second, the C-rich sequences were uncovered by both eRF1-seq (Figure 2D) and MPRA (Figure 4b). Third, only sequences top ranked were selected for the reporter assay. For the positional effects of inserted sequence elements, it is important to note that the proposed mRNA:rRNA interaction is not static because of the continuous mRNA movement along the channel. Instead of using sequences with perfect pairing, we have conducted experiments by placing the C-rich sequences at different positions of the insert. As shown in Figure S3H, the position relative to the stop codon does not seem to matter. In the revised manuscript, we have rephrased several sentences in the main text to avoid confusion.

(6) They attempt to support their model by overexpressing a mutant 18S rRNA with mutations of the penultimate U-U residues to G-G, and present evidence that this decreases the stop codon RPF peaks on mRNAs rich in GA sequences upstream of the stop codons, and has the opposite effect on mRNAs that are C-rich; however, they never indicate the criteria used to assign mRNAs to these two bins, and whether it is based on the GA-rich motifs/LOGOs identified by genome-wide analysis or on the few triplets turned up by the MPRA. Clearly, it would be far better to conduct the same analysis of motif enrichment for high and low pause scores that produced the motif in Figure 1C and determine if the motif for high pausing switches from the GA-rich motif for WT 18S rRNA to a C-rich motif for the mutant, and vice versa for the low pause score mRNAs. It should also be noted that the C-rich sequence used in the reporter can form only 2 base pairs with the mutant 18S rRNA when the mRNA's C-C dinucleotide base pairs with the new G-G dinucleotide in rRNA, but it can actually form 4 base pairs with the WT 18S rRNA sequence in a different pairing register, undermining their interpretation of these data. Note also that there was no analysis done to determine what proportion of 40S subunits actually contain the mutant 18S rRNA, which is expected to be only a minor fraction under the best circumstances, and cannot simply be taken for granted, requiring a direct analysis of the sequences of the 3' ends of 18S rRNA in the cells expressing the mutant 18S.

The Reviewer’s comment on 18S rRNA mutants are insightful. Given the low percentage of ribosomes incorporated with the rRNA mutants, it is not feasible to conduct motif analysis based on ribosome pausing at stop codons. As shown in Figure 5C, stop codon peaks are still evident after 18S mutant transfection albeit less prominent than the wild type. Notably, introducing 18S rRNA mutants into cells is not an easy task, and we have followed closely the protocol published previously (Burman and Mauro. NAR 2012) to obtain meaningful data. We believe (and hope the Reviewer will concur) that the experiment using the 18S rRNA mutants offers critical evidence in support of the mechanism.

(7) They attempt to implicate Rps26 in the pausing by depleting or overexpressing (OE) the protein and comparing pausing at stop codons between the same two ill-defined GA-rich and C-rich bins of mRNAs mentioned above and by assaying the HiBit reporters. Again, they haven't determined whether the amount of Rps26 in mature 40S subunits is reduced or elevated compared to WT cells, and their interpretation of the OE data actually depends on the occurrence of 40S subunits lacking Rps26 in unstressed WT cells, which seems improbable and requires direct confirmation. Also, they haven't quantified the 80S peaks at the stop codons relative to the CDS reads immediately 5' of the stop codons, which varies with Rps26 OE versus the WT control, and doing so might well contradict their conclusion. Moreover, the C-rich and GA-rich HiBiT reporters behave identically rather than oppositely in response to Rps26 OE, which the authors fail to acknowledge or comment on.

The Reviewer might be confused by the role of Rps26 partly due to the lack of clarity in our original description of the results. In yeast, Rps26 can dissociate from fully assembled 80S ribosomes under stress (Yang, et al. Sci Adv 2022). Therefore, although quantifying the Rps26 in mature 40S subunits is informative, it does not infer the composition of 80S ribosomes in cells with Rps26 depletion or overexpression. As pointed out by the Reviewer, we also noticed that, in cells with Rps26 depletion or overexpression, mRNAs with C-rich sequences showed no difference of ribosome density at stop codons. This is quite expected because C-rich sequences have minimal interaction with the 3’ end of rRNA. As a result, Rps26 depletion or overexpression is not supposed to affect ribosome dwell time at stop codons with upstream C-rich sequences. In contrast, only stop codons preceded with GA-rich sequences are influenced by Rps26 heterogeneity. In the revised manuscript, we have clarified this confusion in the main text.

Additional specific comments

(8) In the Summary statement: "We identify a sequence motif upstream of the stop codon that contributes to termination pausing, which was confirmed by massively paralleled": This is unjustified, as the MPRA showed only that a GGG triplet inserted anywhere in 9 nt 5'of the stop codon reduces ribosomes from traversing a stop codon either by blocking leaky scanning or reinitiation after an upstream uORF, and it is unclear why the position of this triplet does not matter nor why other GA-rich sequences capable of base pairing with the 3' end of 18S rRNA were not identified in the MPRA.

As mentioned above, eRF1-seq and MPRA assays are complementary with advantages and disadvantages. Nevertheless, the Reviewer’s comments are well-taken and we have rephrased the Abstract of the revised manuscript.

(9) A supplementary figure explaining EZRA-Seq would be very helpful.

Since EZRA-seq methodology has been published (Mao, et al. NSMB 2023), we think a citation makes more sense. We thank the Reviewer for understanding.

(10) The bottom plots/histograms of Figure 1A are very unclear. What is the y-axis of the bottom histogram, and relative to what elongating ribosomes have been analyzed?

We apologize for the confusion in the histograms of Figure 1A. We stratified all mappable reads into footprints of initiating, elongating, and terminating ribosomes. Like many Ribo-seq results, the majority of footprints are of 29 nt length. If all three ribosome groups are of the same conformation, they are expected to have the same size distribution of the footprint length with the same bar height. It is true for initiating ribosomes (left) but not terminating ribosomes (right). We have now rephrased the figure legend in the revised manuscript.

(11) Page 5: "A close inspection of stop codon footprints revealed an additional peak at -12 nt, which becomes more prominent when the reads are shorter (Figure 1B)." No explanation is offered for this finding. Do forward-shifted termination complexes have an empty A site owing to dissociation of eRF1? If so, they would be undetectable in eRF1-Seq data.

Previous toe-printing assays have shown that eRF1 induces a forward movement of terminating ribosomes, shifting the leading edge from +13 nt to +15 nt (Pisarev, et al. Cell 2007). Moreover, single-molecule analyses have identified distinct pre- and post-termination phases catalyzed by eRF1 (Lawson, et al. Science 2023). Together, these observations suggest that the two 5’ end peaks correspond to pre- and post-terminating ribosome states, with the latter likely adopting a rotated conformation. We have revised the relevant paragraph in the main text.

(12) Page 5: ". It is possible that the two distinct 5' end peaks represent pre- and post-terminating ribosomes, with the latter assuming the rotated conformation. We could not rule out the possibility that these terminating ribosomes have the stop codons at the P-site prior to disassembly." The logic here is difficult to follow.

We have revised the relevant paragraph in the main text.

(13) Figure 1C: provide coordinates relative to the stop codon on this motif.

The motif analysis is position-independent and there is no coordinate on the logo plot.

(14) Page 6: "This was not due to biased downstream sequences as the +4 nucleotide minimally affected the 3'UTR translation (Figure S1C)." The logic here is unclear.

We have rephrased this sentence to “This effect could not be explained by downstream sequence bias, as the identity of the +4 nt had minimal impact on 3’UTR translation (Figure S1C).”

(15) Page 6: "Like Ribo-seq, we also observed a forward shifting of post-terminating ribosomes from eRF1-seq (Figure 2C). " But by definition, they will have eRF1 in the A site, so why are they 26nt vs 29nt?

Like many Ribo-seq results, the majority of footprints are of 29 nt length. However, ribosome populations with smaller footprint sizes are of physiological meanings, likely due to conformation changes.

(16) Page 6 "In agreement with the Ribo-seq data sets, eRF1-seq revealed that not all the mRNAs exhibited eRF1 peaks at the annotated stop codons (Figure 2B), echoing the wide range of termination pausing." It should be determined whether eRF1 occupancy is correlated with 80S occupancy at stop codons in the standard Ribo-Seq. And if not, why?

As shown in Figure 2B, there is a strong correlation between eRF1-seq and Ribo-seq in terms of termination pausing. However, the pausing index will be different between these two data sets due to distinct normalization. We thank the Reviewer for understanding.

(17) Figure 2D: The plot on the left doesn't specify how far upstream the triplets can be from the stop codon. Is the LOGO significantly more similar to that shown in Fig. 1C than expected by chance alone?

In Figure 2D, the codon frequency analysis is position independent. Similarly, the sequence logo in Figure 1C and Figure 2D is also position independent.

(18) Page 7: ". Notably, three different stop codons show similar pausing features and sequence motifs (Figure S1G and S1I)." The figure citations here are incorrect.

We apologize for the missing Figure S1I, which was also pointed out by Reviewer #1. We have now updated Figure S1 in the revised manuscript.

(19) Page 7: The term "false termination" is a poor descriptor if termination doesn't occur.

We have followed the Reviewer’s suggestion by replacing “false termination” with “failed termination”.

(20) Page 8: "Consistent with previous reports 27, mutating the stop codon UAG abolished the reinitiation event that drives out-of-frame HiBiT translation (Figure 3E)." How is HiBit assayed? No details are given in the legend. This result doesn't confirm any of the actual eIF1 peaks upstream of stop codons, just that REI can occur at some level 5' of stop codons; and the eRF1 peak at the HiBit stop codon would be 3' of the peak at the main stop codon.

HiBiT assay is a standard reporter like luciferase and Promega offers a detection kit, as described in the methods section. The result shown in Figure 3E is to confirm stop codon-associated reinitiation, which suggests that ribosomes migrated from the stop codon could contain eRF1 before reaching a start codon for reinitiation. We have revised this paragraph to avoid confusion.

(21) Figure 4A: Unclear what position 0 to 6 in the bottom heat map corresponds to in the inserted 9 nt sequences. Are these codon positions vs. nucleotide positions? The legend lacks explanatory information.

Figure 4A shows nucleotide positions (x axis) grouped by 3nt to reflect codon information (y axis). For the inserted 9nt random sequences, the last two nucleotides cannot be used because of the fixed nucleotides downstream of the insert. The same analysis has been reported in our prior study (Jia, et al. NSMB 2020).

(22) Page 8: "For instance, codons enriched in frame 2 belong to NUA and NUG, another indication of in-frame stop codons (Figure S3B, bottom panel). " Need more or better explanation here.

We have rephrased this sentence in the main text. “Codons enriched in alternative reading frames were also informative; for example, codons enriched in frame 2 predominantly belong to NUA and NUG, consistent with frameshifted presentations of in-frame stop codons (Figure S3B, bottom panel).”

(23) "This is likely due to the faster turnover of these mRNAs because of 3'UTR translation". Need more or better explanation here.

MPRA in Figure S3C showed that mRNA variants containing C-rich downstream sequence were depleted from both monosome and polysome fractions. Since 3’UTR translation is well-established to induce mRNA decay, it is possible that these sequences are under-represented due to mRNA turnover. We have added more explanations in this paragraph in the revised manuscript.

(24) " Figure 4B: The logic and assumptions of this assay are not explained. How do ribosomes traverse the uORF, by leaky scanning or by stop codon read-through that is impeded by a ribosome stalled at the uORF stop codon? Presumably, it can't be read through as the uORF is out of frame and translation would likely terminate quickly.

The rationale of Figure 4B is very similar to Figure 4A, except for the presence of the stop codon UAG. Under efficient termination, a monosome enrichment is expected, which could be promoted by termination pausing or structural hinderance by G-rich sequences. In contrast, stop codon readthrough or reinitiation would lead to polysome enrichment. We have thoroughly revised this paragraph in the main text.

(25) Figure 4B results: It's unclear why M/P ratios are so low in Figure 4B vs Figure 4A as all constructs in 4B contain a stop codon and should have the high M/P ratios seen for the constructs in panel (A) with stop codons inserted. It's also unclear why the high M/P ratio should be so limited to GGG triplets vs. other triplets that conform to the GA-rich motifs identified above, and also why this triplet would not function at codon position 6. Similarly, it's unclear why only CCG and CCC and not CCU and CCA have an effect, and why only 3 of 9 codons with 2 or more C's have the effect, all suggesting that specific sequences and not just C-rich sequences are promoting read-through. Yet, no C-rich motif was discernible in the profiling experiments above.

We appreciate the Reviewer’s careful reading of our manuscript. In profiling experiments shown in Figure 2, we did observe C-rich codons albeit with variations. Possible reasons include sequence differences between human genome and randomized sequence combinations. In addressing the Reviewer's question 23, we have thoroughly revised this paragraph in the main text.

(26) Page 9: "These results are in line with the sequence specificity in termination pausing revealed by Ribo-seq and eRF1-seq." This is unjustified as the results in 4B are restricted to only GGG triplets rather than numerous triplets that equally conform to the AAGAAGA motif defined above.

We apologize for the overstatement in this sentence. In addressing the Reviewer's question 23 and 24, we have thoroughly revised this paragraph in the main text.

(27) Page 9: "This result is congruent with the MPRA assay, suggesting that the C-rich coding sequence preceding the stop codon not only reduces termination pausing, but also promotes downstream translation." This is unjustified as the single C-rich sequence chosen for the analysis in Figure 4C is not representative of the two C-rich triplets identified in Figure 4B, showing strong evidence of read-through.

In Figure 4C, both C-rich and GA-rich sequences were chosen from shared elements between eRF1-seq and MPRA as they represent physiological sequences associated with termination pausing. The reporter assay is crucial in linking the lack of termination pausing with 3’UTR translation. We thank the Reviewer for understanding.

(28) The analyses in Figures 4C-D suffer from a lack of the no-stop codon controls to allow the standard quantification of read-through as a percentage of continuous translation in the zero frame in the absence of a stop codon.

The Reviewer might have missed the no-stop codon control in Figure 4C, which contains reporters with (bottom) and without (top) UAG stop codon. In Figure 4D, it is not feasible to include no-stop codon control for frameshifting reporters as the HiBiT value will be out-of-chart several orders of magnitude.

(29) Page 10: "Therefore, the C-rich coding sequence triggers ribosome sliding at the stop codon, resulting in 3'UTR translation in all three reading frames." Sliding is an imprecise term. It is presumably a stop codon readthrough accompanied by frameshifting.

We agree with the Reviewer’s suggestion and have replaced the word of “sliding” with “readthrough”.

(30) Page 10: The citation to Figure S3H is incorrect, as there is no panel H.

We are glad to have this opportunity to fix this error. We have now added panel H into the Figure S3 in the revised manuscript.

(31) Page 10: "When the ribosome occupancy in the CDS was normalized, loss of ABCE1 led to a modest increase of stop codon peaks (Figure S4C)". Is this increase reproducible in replicates and statistically significant, as it seems very slight?

The increased ribosome peak at stop codons in cells lacking ABCE1 is not significant, partly due to incomplete depletion of ABCE1 as shown in Figure S4A. Since ABCE1 is not the focus of this study, we did not attempt to knock out ABCE1, which could cause cellular toxicity.

(32) Page 11: "Notably, the elevated ribosome density occurred at all stop codons, an indication of global effects." Where are the data substantiating this claim?

We apologize for the confusion here. In the revised manuscript, we have deleted this sentence from the main text.

(33) Page 11: "A closer look revealed that silencing ABCE1 increased the ribosome density at the -15 nt position". This claim is not convincing in the 29 nt read data, where it should be observed.

We agree with the Reviewer that the increased ribosome density at the -15 nt position is more evident for shorter footprints. We have revised the sentence in the main text.

(34) Page 11: "Since the 3' end of 18S rRNA contains a highly conserved U-rich sequence (GAUCAUUA), the GA- rich sequence element of mRNA could follow U:A and U:G base pairing near the exit site" (Figure 5A and S5A). By contrast, the C-rich sequence motif on mRNA would escape the 18S rRNA checkpoint, resulting in faster mRNA passthrough." This seems simplistic, as there would also be three G-A or A-G mispairings with 18S rRNA at other positions of the (G/A)AAGAAGA motif. Also unclear what the C-rich motif actually is, making it impossible to determine how many pairings it could make with the 18S rRNA sequence.

Unlike base pairing on RNA structures, the putative rRNA:mRNA interaction is dynamic because of the continuous movement of mRNA along the ribosome channel. In fact, perfect base pairing might not be instrumental. Therefore, the difference between GA-rich and C-rich sequences is reflected in the accumulated effect. As mentioned above, the C-rich sequences are derived from both eRF1-seq and MPRA.

(35) Figure S5B: Showing this sequence is misleading. While not described, it is presumably the DNA sequence of the plasmid, not the rRNA sequence, as there is 100% of the mutant sequence. They need to sequence the 3' end of rRNA isolated from ribosomes to confirm the presence of mutant ribosomes at appreciable levels.

The Reviewer is correct that the sequences shown in Figure S5B are from the plasmids. To avoid such confusion, we have removed the sequences in the updated Figure S5B.

(36) Page 12: "When mRNAs are stratified based on the sequence motif upstream of stop codons, we found that overexpression of the 18S mutant reduced the differential termination pausing between GA-rich and C-rich sequences (Figure 5C)". It is not explained what GA-rich or C-richness means precisely. Moreover, the same kind of analysis done in Figure 1C should have been conducted here to determine the LOGOs for high and low pausing for WT vs mutant 18S rRNA.

We understand why the Reviewer repeatedly ask about the GA-rich and C-rich sequences, partly due to the lack of clarity in our original description of the analysis. The GA-rich transcripts were defined as those have the upstream 15-nt sequence with G or A nucleotides more than 65% (9 nt); whereas C-rich transcripts were defined as those with C more than 40% (6 nt). We have now updated the methods section in the revised manuscript.

(37) Page 12: "Notably, the 3' end sequence of 18S rRNA is highly conserved (Figure S5D)". There is no Figure S5D in the figures.

We are glad to have this opportunity to fix this error. We have now added panel D and E into Figure S5 in the revised manuscript.

(38) Page 13: "Further supporting the sequence specificity of termination pausing, testis mRNAs with prominent stop codon peaks are enriched with GA-sequences upstream of the stop codon (Figure S6C). The same group of mRNAs, however, barely exhibit termination pausing in liver." Again, motif analysis of high and low pausing should have been done here.

The motif analysis in mouse tissue samples is less informative because GA-rich sequences will be over-represented in testis, whereas the same group will be under-represented in liver. We had to select the shared mRNAs for comparative analysis. We thank the Reviewer for understanding.

(39) Page 13: "While liver exhibited a similar distribution of Rps26 and RACK1 in polysome fractions, testis showed an evident depletion of Rps26 in polysome (Figure 6C). Notably, a substantial amount of Rps26 is present in the ribosome-free fraction of testis." They failed to normalize Rps26 levels in polysomes for bulk polysome levels, as indicated by the A260 tracings to determine if polysomes are depleted of Rps26, or rather, there is less polysomal Rps26 simply because polysomes are less abundant.

We agree with the Reviewer’s notion regarding different polysome traces between testis and liver. Because the polysome volume is difficult to normalize, we used RACK1, a constitutive component of ribosome, to quantify the amount of polysome.

(40) Page 14: "Indeed, normal mode analysis (NMA) by anisotropic network models suggests that, in the absence of Rps26, both the -3 to -9 extension of the mRNA and the 3' end of 18S rRNA can twist and approximate to each other with improved mutual parity (Figure 7B)." It is unclear what this means.

Normal Mode Analysis (NMA) by Anisotropic Network Model (ANM) is a coarse-grained computational method used to study biomolecular dynamics by modeling proteins as a network of nodes connected by springs. Unlike the Gaussian Network Model (GNM), ANM calculates the full 3D directional preference of motion, enabling characterization of conformational changes, domain movements, and flexibility in large macromolecules. We have added a citation (Bahar, I. et al. 2005) in the revised manuscript.

(41) Page 14: "To investigate whether Rps26 haploinsufficiency affects ribosome dynamics at stop codons, we knocked down Rps26 from HEK293 cells using shRNA (Figure S7A)". Haploinsufficiency properly refers to a heterozygous null/WT genotype, not shRNA knockdown.

The Reviewer is correct in terms of haploinsufficiency. We have replaced the word of “haploinsufficiency” with “reduced Rps26 levels” in the revised manuscript.

(42) Page 14: "The reciprocal change echoes the tissue-specific differences in initiation and termination (Figure 6A). " It's unclear why these peaks should be reciprocally related mechanistically, so examining changes in their ratio may not be incisive. Rps26 KD could reduce the efficiency of termination independently of pausing. And does Rps26 KD affect eRF1 occupancies in parallel with 80S occupancies?

A prior study reported that Rps26 regulates translation initiation by recognizing Kozak sequence elements (Ferretti, et al. NSMB 2017). We therefore speculate that the role of Rps26 in termination might be correlated, although we don’t have direct evidence. We have further clarified this point in the discuss section of the revised manuscript.

(43) Page 14: "The increased termination pausing, once again, primarily occurs at stop codons preceded by GA-rich sequences (Figure 7C)". No statistical analysis of replicates was done to see if the increase is significant, as it is quite small. They could have stratified mRNAs according to the number of base-pairs they can form with 18S rRNA rather than using this nebulous GA-richness, and see if the conclusion still holds.

The metagene analysis shown in Figure 7C is standard for comparison of ribosome footprint distribution. We agree that the increase of termination peak at stop codons preceded by GA-rich sequences is not as striking as it should be, this is an underestimate because only a small fraction of ribosomes have sub stoichiometry of Rps26.

(44) Page 14: "Remarkably, when mRNAs are stratified based on the sequence motif upstream of stop codons, we found that overexpression of Rps26 reduced the ribosome density (>50%) at stop codons preceded by the GA-sequence (Figure 7E)." They failed to normalize reads to the CDS occupancies to control for fewer ribosomes reaching the stop codons, especially considering that depletion of elongating 80S appeared to occur just upstream of stop codons on Rps26 OE. The same problem exists for the C-rich mRNAs. Also, their interpretation of the effects of Rps26 OE depends on there being Rps26-lacking 40S subunits in WT unstressed cells, which seems unlikely and has not been established directly. Finally, they didn't show increased Rps26 content in 40S subunits on Rps26 OE, which is also required.

This question is the same as #7, which we have fully addressed in this letter (page 7).

(45) Page 15: "To affirm the mechanistic connection between stop codon pausing and termination fidelity, we conducted HiBiT reporter assays that showed increased 3'UTR translation in cells with Rps26 overexpression (Figure 7F)." But both the C-rich and GA-rich reporters show increased expression on Rps26 OE. Why should that be if the C-rich sequences don't base pair with 18S rRNA in WT cells and are unaffected by Rps26 depletion? These data suggest that some other mechanism underlies the increased expression of the GA-rich reporters seen on Rps26 OE.

The Reviewer’s concern is valid, and we agree that additional mechanisms might contribute to the increased reporter expression. The simplest explanation is that Rps26 overexpression promotes ribosome biogenesis, which globally increases mRNA translation. Supporting this notion, more polysome could be observed in cells with Rps26 overexpression (Figure S7E).

(46) Page 15: "Without pausing at stop codons, terminating ribosomes are likely to undergo incomplete dissociation, resulting in continuous translation in 3'UTR." The language here is imprecise. Are they proposing reinitiation by unrecycled 80S ribosomes, or stop codon read-through with or without frameshifting, or both?

This question is the same as #2, which we have fully addressed in this letter (page 3).

(47) Page 15: "Importantly, lack of termination pausing leads to stop codon-associated random translation, giving rise to mixed C-terminal extension." Again, what does this mean? Read-through generally accompanied by frameshifting?

Stop codon-associated random translation differs from ribosome readthrough, reinitiation, or frameshifting. We have extensively clarified this confusion in the revised manuscript.

(48) Page 16: "For terminating ribosomes, the prolonged dwell time at stop codons offers an extended window for eRF1 loading, peptide cleavage, and ribosome recycling." This sentence is confusing because the eRF1-Seq data suggest that the pause occurs after eRF1 decodes the stop codon, with delayed peptide cleavage and recycling.

We thank the Reviewer’s effort to improve our manuscript. We have rephrased the entire paragraph in the revised manuscript.

**Reviewer #3 (Recommendations for the authors):**

The manuscript is well-written, and the conclusions are overall well-supported by the data. I have only a few relatively minor questions and comments:

(1) For termination sites overlapping with coding regions, the lack of 3-nt periodicity downstream of these sites could result from overlapping translation of multiple ORFs, rather than indicating that translation readthrough events can happen in multiple frames. Could the authors clarify this interpretation?

We appreciate the Reviewer’s positive comments on our manuscript. The Reviewer is correct that overlapping ORFs would result in the lack of 3-nt periodicity. Although it is common for overlapping ORFs near the canonical start codons, ORFs overlapping the canonical stop codons are rare. Nevertheless, we have rephrased the statement in the revised manuscript.

(2) The observation that multiple eRF1-seq peaks are located within CDS regions suggests that eRF1 may compete with A-site tRNAs during elongation. This is an interesting finding. Do the authors think this competition could lead to premature termination, or is it more likely to represent elongation pausing? Additionally, do the authors observe corresponding ribosome pausing peaks at these sites in conventional Ribo-seq data?

The Reviewer’s comment on eRF1-seq peaks in CDS is insightful. We agree that pre-mature termination is possible because of competition. However, we do not observe corresponding ribosome pausing peaks in regular Ribo-seq, presumably due to low frequency of which events.

(3) Regarding the regulation of ribosome pausing across tissue types, how robust are these results? For example, are the tissue-specific effects (such as stronger pausing in the testis) consistent among different mice or across age groups, given that many aspects of translational regulation are known to change with aging?

We found that tissue-specific distribution of ribosome footprints is highly reproducible, especially liver and testis. Notably, the lack of termination peaks in liver is also reported by other independent studies (Gobert, et al. PNAS 2020), arguing that such effect is not a result of sequencing bias. We haven’t compared mice with different ages, but aging-associated translational regulation is an interesting topic awaits further investigation.

**Reviewer #4 (Recommendations for the authors):**

(1) Translation termination has been studied by ribose in several organisms, including mammalian cells and yeast. In those cases, what is analyzed is not the peak height at the stop codon, but rather the difference in the ribosome density before and after the stop. Thus, analyzing peak height is not validated. I understand that this is relevant only for the ribosome profiling experiments (and Ezra-seq), not the RF1 profiling. But the large majority of the data was acquired that way.

With due respect, we disagree with the Reviewer’s point regarding how to study ribosome dynamics at stop codons. Comparing footprint density before and after stop codons does not infer dynamics of terminating ribosomes. By establishing eRF1-seq, we are for the first time able to analyze ribosome behaviors at stop codons, which represents a significant advancement of technological development.

(2) Moreover, the data do not reproduce previous findings, and no attempt is made to connect them to previous data. Previous data have shown that stop codon efficacy varies. This is not reproduced (S1C). Similarly, an effect from the +1 residue is not reproduced. The data isn't stratified by different stop codons, and previous work has shown that different surrounding residues have different effects in the context of different stop codons. Thus, none of the sequencing data is validated or trusted and does not reproduce previous findings.

We are certainly aware of previous findings regarding stop codon readthrough. We would like to emphasize that our findings do not contradict established principles of translation termination. Rather, enabled by the development of eRF1-seq, we provide new insights into termination dynamics that extend existing models.

(3) The GA-rich sequence identified by Ezra-Seq and RF1 seq is not the same, and it differs from previous sequences (Wangen &Green).

We don’t quite understand why the Reviewer is preoccupied with prior studies without accepting new results obtained from newly developed technology. The GA-rich sequences identified by Ezra-Seq and eRF1-seq are similar, albeit not identical. This is simply because eRF1-seq offers much higher resolution to reveal termination pausing than regular Ribo-seq.

(4) The authors claim that the majority of Rf1 peaks are at stop codons, but that is not true. It is only about 30% of the peaks. Also, not all mRNAs have peaks at the stop codons. That is, at best, problematic. Finally, there are mRNAs that are known to "suffer" from NMD. What do these look like in the Ezra-Seq and RF1-Seq? How about mRNAs that have programmed frameshifts? The eRF1 data is invalid.

The Reviewer is confused about the eRF1 peak density versus frequency, which has totally different meanings. Additionally, the Reviewer seems to be surprised that not all mRNAs have peaks at the stop codons. The differential ribosome dynamics at stop codons is an exciting feature previously unappreciated, rather than problematic. Regarding programmed frameshifting, we argue that such events are rare in mammalian cells.

(5) Figure 4 has many flaws; it is hard to know where to start. First, instead of the M/P ratio, one should analyze M/M+P, to normalize out differences in the loading and effects from collisions, which are guaranteed to occur here, but not considered or analyzed. Second, the data are analyzed as if what matters are codons in the P and E site (and beyond, where there are definitely NOT recognized codons). While there is evidence for some interactions, one would think that an additional analysis based on sequence would be helpful. Also, the supplemental data indicate that very rarely are there reciprocal changes (as should be the case), as seen for stop codons. Thus, the assay is at best questionable and likely worse.

The Reviewer appears to be unfamiliar with massively parallelled assay, which has been widely used to uncover sequence elements crucial in translational regulation. We urge the Reviewer to read our prior study using MPRA to investigate alternative translation initiation (Jia, et al. NSMB 2020). The similar approach has also been used to decipher 5’ UTR sequence elements in mRNA engineering (Sample, et al. Nat Biotech 2019).

(6) Things do not look up for the HiBit reporter assay. The two sequences clearly have effects on translation without considering stop codon context (Figure 4C), which need to be taken into account. Also, the effect from the sequences varies in the context of the assay in 4C and 4D (2-fold vs. 5-fold), further questioning the assay. Moreover, the authors claim that re-initiation cannot account for Hibit levels, but that is clearly incorrect. The western in Figure 4E does not reproduce the data in 4D. While Hibit goes up as in 4D, the putative GFP-fusion goes down. Finally, while the second reading frame should be more efficient, it is not explained and further argues for an artifact. Previous work (and work herein) suggests that read-through occurs equally in each reading frame.

The Reviewer is confused about the HiBiT-based reporter assay shown in Figure 4C-4E. First, we have included important controls, i.e., same reporters without stop codons, to normalize sequence variation. Second, Figure 4C and 4D used totally different reporters and it is not appropriate to directly compare their values. Third, re-initiation events would not generate fusion proteins containing the N-terminal GFP. The Reviewer is encouraged to re-examine the results presented in Figure 4.

(7) No controls for these assays are presented: e.g., stimulation by antibiotics, ABCE1 depletion, etc.

We are not sure which assay the Reviewer is referring to. For reporter assays shown in Figure 4, we focused on effects of *cis*-sequence elements, rather than *trans*-acting factors. We thank the Reviewer for understanding.

(8) Figure 5 has similar problems. I don't understand how Figure 5A is made, but when one overlays the cited structures on Rps26, the molecules are identical. I guess the authors chose to build non-existing sequences differently into the structure. There is no basis for that. In panel C, and the same in Figure 7, the number of analyzed mRNAs varies. This could influence the outcome, and the EXACT same set of mRNAs should be analyzed. But the main problem here is that the authors need to analyze readthrough and not peak height, as detailed above. Essential controls are missing that show what fraction of the 18S rRNA is mutated. Previous work has shown that 2 nt-truncated 18S rRNA is actively degraded. It is hard to believe how 15% of altered ribosomes can abolish 100% of the effect from the C-rich sequences. Important validation is missing: the authors should analyze rRNA sequences in their ribo-seq dataset to demonstrate that they have the mutated rRNAs, and that these enrich and de-enrich as predicted.

The Reviewer’s comment on Figure 5A is baseless. As indicated in the Figure legend, Figure 5A was made from the existing cryoEM structure (PDB: 6ZMW). Regarding 18S rRNA mutants, we simply followed prior studies (Burman and Mauro. NAR 2012) and there is no evidence indicating degradation of such rRNA mutants. Given the low percentage of ribosomes incorporated with the rRNA mutants, the observed effect on termination pausing represent an underestimation, rather than an overstatement.

(9) In Figures 5-7, the authors develop a model that the sequence selectivity arises from base pairing between 18S rRNA and the mRNA. If so, then they should really stratify the data by the number of WC pairs that can be formed. And only WC pairs, as GU pairs have a totally different geometry that will likely be discriminated against in this context. Also, the mutation is in a part of the helix that has no effect (Figure S3G). Thus, the data within the manuscript are inconsistent.

As the Reviewer might be aware, GU pairs are commonly found in tRNA and rRNA structures. Since both WC and GU pairs contribute to mRNA:rRNA interaction, there is no point to stratify sequences based on different pairing format. Additionally, we would like to point out that the putative mRNA:rRNA interaction is not static, considering the continuous movement of mRNA along the ribosome channel.

(10) Figure 6 does not agree with published data (Li et al., Nature 2022). Previous work did not show testis depletion of Rps26 in purified ribosomes. This is the critical difference, as the authors here did not purify ribosomes. Also, another Rps is an essential control, even if purified ribosomes are used. This dataset should not be shared. Depletion from polysomes is hard to believe, as overall, there is less signal in the polysomes.

The Reviewer finally made a good point regarding Rps26 in testis. In our study, we did not separate different cell types such as spermatocytes and therefore we do not know which cell type dominantly influences termination pausing.

Regarding varied Rps26 levels in different tissues, we noticed different polysome between testis and liver. Because the polysome volume is difficult to normalize, we used RACK1, a constitutive component of ribosome, to quantify the amount of polysome.

(11) Figure 7 has similar problems to Figure 5. Different pools of mRNAs are analyzed; peak height is not validated. Overexpression of Rps26 is not shown, as only Myc is shown, not Rps26. Beyond that, increased occupancy in ribosomes needs to be shown for the effect to come from ribosomes. Given how sick the cells are, it is most likely that all effects are secondary and arise from whatever else is going on in the overexpression or depletion of Rps26. No controls are presented to show specific effects from Rps26.

We are surprised that the Reviewer ignored the supplementary data that shows Rps26 levels. Regarding controls, it is not appropriate to use different ribosomal proteins because every ribosomal protein has its won functionality. We acknowledge that experiments by gene knockdown is not perfect, but the results are still informative especially when different mRNA pools from the same cells are compared.

(11) The authors need to check Rli1/ABCE levels in their cells. Their data have features that are indicative of low ABCE1 levels. These include a very small effect from ABCE1 depletion. These could be responsible for some of the effects they observe.

Once again, we are surprised that the Reviewer ignored the supplementary data that already shows ABCE1 levels in cells with or without ABCE1 knockdown (Figure S4A). Constantly addressing the Reviewer’s lack of careful reading of our manuscript is frustrateing. Nevertheless, we have thoroughly revised the entire manuscript by clarifying interpretations, moderating mechanistic claims, and expanding relevant discussion.
